# Phlebotomine sand fly distribution and abundance in France: A systematic review[Fn FN1]

**DOI:** 10.1051/parasite/2024045

**Published:** 2024-08-07

**Authors:** Jorian Prudhomme, Jérôme Depaquit, Florence Robert-Gangneux

**Affiliations:** 1 Université de Rennes, Inserm, EHESP, Irset (Institut de Recherche en Santé Environnement Travail), UMR_S 1085 35000 Rennes France; 2 Université de Reims Champagne-Ardenne, Faculté de Pharmacie, UR EpidémioSurveillance et Circulation de Parasites dans les Environnements (ESCAPE), and ANSES, USC Pathogènes-Environnement-Toxoplasme-Arthropodes-Réservoirs-bioDiversité (PETARD) Reims France; 3 Centre Hospitalo-Universitaire, Laboratoire de Parasitologie-Mycologie 51092 Reims France

**Keywords:** Systematic review, PRISMA, Sand flies, Distribution, France

## Abstract

Global changes in climate are contributing to modified Phlebotomine sand fly presence and activity, and the distribution of the pathogens they transmit (e.g., *Leishmania* and Phlebovirus), and are leading to their possible extension toward northern France. To predict the evolution of these pathogens and control their spread, it is essential to identify and characterize the presence and abundance of potential vectors. However, there are no recent publications describing sand fly species distribution in France. Consequently, we carried out a systematic review to provide distribution and abundance maps over time, along with a simplified dichotomous key for species in France. The review adhered to PRISMA guidelines, resulting in 172 relevant capture reports from 168 studies out of the 2646 documents retrieved, of which 552 were read and 228 analyzed. Seven species were recorded and categorized into three groups based on their abundance: low abundance species, abundant but little-studied species, and abundant vector species. Sand flies are certainly present throughout France but there is a greater diversity of species in the Mediterranean region. *Phlebotomus perniciosus* and *Ph. ariasi* are the most abundant and widely distributed species, playing a role as vectors of *Leishmania*. *Sergentomyia minuta*, though very abundant, remains under-studied, highlighting the need for further research. *Phlebotomus papatasi*, *Ph. perfiliewi*, *Ph. sergenti*, and *Ph. mascittii* are present in low numbers and are less documented, limiting understanding of their potential role as vectors. This work provides the necessary basis for comparison of field data generated in the future.

## Abstract (fr)

Les changements globaux du climat contribuent à modifier la présence et l’activité des phlébotomes, ainsi que la répartition des pathogènes qu’ils transmettent (par exemple *Leishmania* et Phlebovirus), et conduisent à leur éventuelle extension vers le nord de la France. Pour prédire l’évolution de ces pathogènes et contrôler leur propagation, il est essentiel d’identifier et de caractériser la présence et l’abondance des vecteurs potentiels. Il n’existe cependant aucune publication récente décrivant la répartition des espèces de phlébotomes en France. Par conséquent, nous avons réalisé une revue systématique pour fournir des cartes de répartition et d’abondance dans le temps, ainsi qu’une clé dichotomique simplifiée pour les espèces françaises. La revue a respecté les lignes directrices PRISMA, aboutissant à 172 rapports de capture pertinents provenant de 168 études sur les 2 646 documents récupérés, dont 552 ont été lus et 228 analysés. Sept espèces ont été recensées et classées en trois groupes en fonction de leur abondance : les espèces de faible abondance, les espèces abondantes mais peu étudiées et les espèces vectrices abondantes. Les phlébotomes sont certes présents partout en France mais on trouve une plus grande diversité d’espèces dans le bassin méditerranéen. *Phlebotomus perniciosus* et *Ph. ariasi* sont les espèces les plus abondantes et les plus largement réparties, jouant un rôle de vecteurs de *Leishmania*. *Sergentomyia minuta*, bien que très abondant, reste sous-étudié, ce qui souligne la nécessité de recherches plus approfondies. *Phlebotomus papatasi*, *Ph. perfiliewi*, *Ph. sergenti* et *Ph. mascittii* sont présents en faibles nombres et sont moins documentés, ce qui limite la compréhension de leur rôle potentiel en tant que vecteurs. Ce travail fournit la base nécessaire pour la comparaison des données de terrain générées à l’avenir.

## Introduction

Phlebotomine sand flies (Diptera, Psychodidae) are small insect vectors of pathogens such as *Leishmania* and Toscana virus (Phlebovirus). These pathogens are endemic in the Mediterranean region and are responsible in mainland France for cutaneous and visceral leishmaniasis (due to *Leishmania infantum*) [39] and febrile illnesses and meningitis or encephalitis, respectively [8]. In the south of France, a mean number of 22.6 cases of autochthonous leishmaniasis are recorded annually [39], and Toscana virus (TOSV) is one of the most prominent causes of aseptic meningitis during the warm season [8]. Importantly, most individuals remain asymptomatic after infection [29–31], which clearly results in an underestimation of the circulation of these pathogens on the territory.

Additionally, climate change, together with the intensification of international trade, contribute to modify vector life-history traits (e.g., intensified sand fly presence and activity) and pathogen distribution, and lead to the extension of these pathogens towards northern France. These changes could extend the geographic reach of some vector species (e.g., *Phlebotomus perniciosus*) or bring pathogens and new potential vectors into contact (e.g., *Phlebotomus mascittii*, whose vector competence for *Leishmania* and TOSV is suspected [32, 36]). However, no recent publications have provided an update on sand fly presence and species distribution in France. Nevertheless, to predict the evolution of sand fly-borne virus (SFBV) and to control spread, it is essential to identify and characterize the presence and abundance of potential vectors.

In this context, we carried out a systematic review of the literature reporting the presence of sand fly species in mainland France (including Corsica). This work is part of the European CLIMOS project (Climate Monitoring and Decision Support Framework for Sand Fly-borne Diseases Detection and Mitigation, https://climos-project.eu/), which aims to assist mitigation of climate and climate change-induced emergence, transmission and spread of vector-borne and zoonotic pathogens. Therefore, this work aimed to estimate the different sand fly species distribution and abundance, according to the data reported in the literature, in order to provide a basis for future comparison of data delivered by this European project.

## Methods

### Search strategy

We conducted a systematic review with no publication date, but with language restrictions, following the Preferred Reporting Items for Systematic Reviews and Meta-Analyses (PRISMA) reporting checklist [37, 38] as previously described [43]. The literature search was carried out in two databases: PubMed and Web of Science. These databases were selected according to systematic review recommendations [19] and the following criteria: subject, number of accessible documents, boolean and parenthesis functional, and bulk download. The search terms used were the following: (France OR Corsica OR French) AND (sandfl* OR “sand fl*” OR phlebotomin*). The last search was performed on the October 26, 2023. Additional documents from identified references and from the following French sand fly experts were added and analyzed under the same process: Depaquit J. (Université de Reims-Champagne Ardennes, Reims, France), Gantier J-C. (Université Paris-Saclay, Paris, France), Prudhomme J. (Inserm, Rennes, France), Rahola N. (Institut de Recherche pour le Développement, Montpellier, France), and Schaffner F. (University of Zurich, Zurich, Switzerland).

### Screening process and study selection

The documents identified were exported and analyzed in CADIMA [27, 43]. After removal of duplicates, an initial review based on title and abstract, or only title if abstract unavailable, was performed. The document was processed for full text reading if the inclusion criteria were not certain from the title and abstract. Exclusion criteria were defined as: (a) publication not concerning France; (b) research not based on sand flies; (c) document without entomologic indicators (presence or abundance); or (d) publications in a language other than English or French. Each chapter or article was reviewed individually as described above for books or whole journal issues selected. Documents selected for full reading were collected using the above-mentioned databases or through the authors or journals directly. The gathering of documents was achieved in March 2024. For full reading, each publication was reviewed for inclusion according to the same eligibility criteria as for title and abstract. As a quality control measure, excluded articles were reviewed a second time.

### Data extraction and analysis

A qualitative analysis was conducted to account for the wide variety of publication styles and research methods presented. From the studies included, data were extracted to determine: (a) the species; (b) their presence or abundance (if available); (c) their spatio-temporal distribution; (d) the trapping method; (e) the duration of the study; (f) the trapping period; (g) the GPS coordinates; and (h) the altitude. One person (JP) performed data extraction and analysis. Graphics were created with Prism Software, version 9. For each sand fly species, distribution maps (presence and abundance) were generated using the website of the Institut National de l’Information Géographique et Forestière (IGN) [23].

## Results

### Bibliometric data

**Figure 1:**
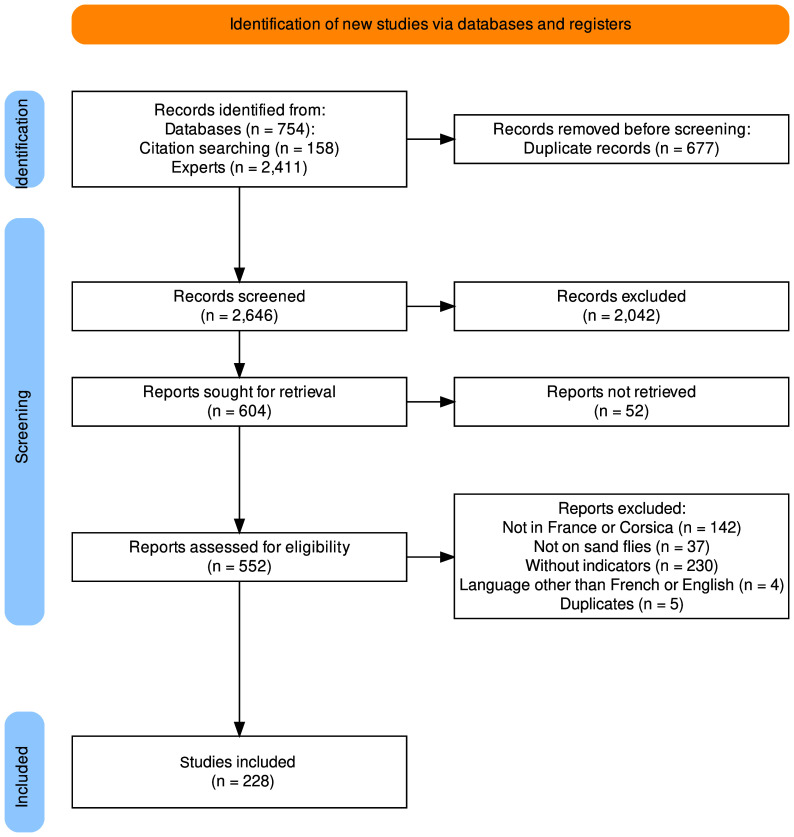
PRISMA flow chart of study selection process.

The full search retrieved 3323 documents (2411 from experts, 754 publications from identified databases, and 158 by publication bibliography cross-check). We removed 677 duplicate documents. A total of 2646 documents were reviewed, of which 2042 documents were excluded for the following reasons: the study was (a) not carried out in France or Corsica (*n* = 1550); (b) not based on sand flies (*n* = 669); (c) written in a language other than English or French (*n* = 59); or (d) without entomologic indicators (presence or abundance) (*n* = 868). Of the 604 remaining documents, 52 were not accessible. A total of 552 documents were entirely read and 324 were further excluded according to the inclusion criteria (Fig. 1). Of the 228 remaining studies, 60 were excluded as no primary data were presented. However, among these 60 documents, 3 that did not present original data but detailed primary captures of non-accessible articles previously identified (*n* = 5) were retained. Finally, 168 studies were included, comprising 172 capture description reports, which were extracted and analyzed. The full list of documents is provided in Supplemental Table 1. The screening process detailed in the PRISMA flow diagram is available in Figure 1 and the characteristics of studies included are summarized in Table 1.

**Table 1:** Characteristics of included studies according to their publication dates.

	<1920	1921–1930	1931–1940	1941–1950	1951–1960	1961–1970	1971–1980	1981–1990	1991–2000	2001–2010	2011–2023	Total (%)
Number of extracted reports	13	11	26	16	15	11	19	24	8	13	16	172 (100)
Reports on sand fly presence	10	10	17	8	6	6	1	11	5	8	10	91 (52.9)
Reports on sand fly abundance	3	1	10	8	9	5	18	13	3	5	6	81 (47.1)

### Non-analyzable recorded data

Recording environmental and capture factors is critical for studying sand flies. These factors influence their behavior, distribution, and abundance, making accurate documentation essential for understanding ecological dynamics. However, in our study, the absence of such data limits our ability to comprehensively analyze the impact of these factors on sand fly populations.

Regarding the GPS coordinates, only 11% (*n* = 19/172) of the references provide this information. Similarly, only 50 (29%) and 62 (36%) documents provided altitude data (ranging from 10 to 1420 meters) and the duration of the study (mean 24.6 days ± 23.9), respectively. The lack or inconsistency of these data rendered it impossible to ascertain whether these factors statistically impact the occurrence and/or abundance of sand flies. Moreover, as there are many differences in the research questions and the captures covered different scales, distributions and species, it is not possible to analyze these data or arrive at conclusions relevant to the whole territory.

Concerning the trapping period, the month of capture was described for 79.7% of documents (*n* = 137/172). The majority of captures were made during the sand fly activity period, i.e., from June to August (*n* = 128/137, 93.4%). The season of capture was often described (*n* = 144/172, 83.7%), and was almost always in summer (*n* = 142/144, 98.6%). These results are consistent with the existing knowledge base on sand fly ecology [2].

### Sand fly species recorded

**Figure 2:**
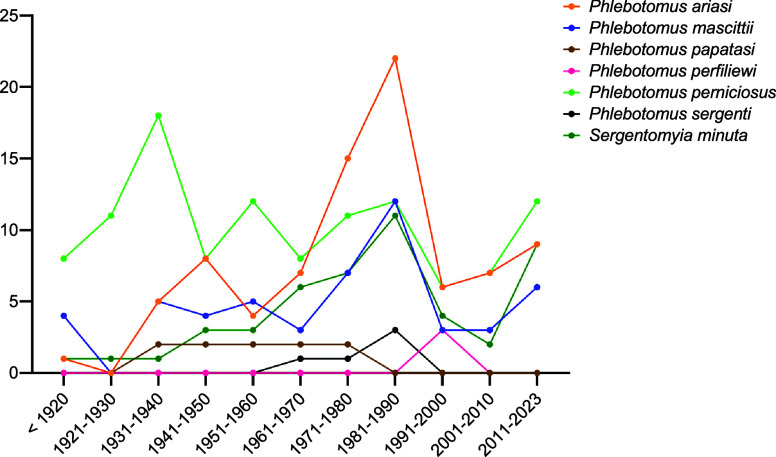
Number of studies by species according to the year of publication.

**Figure 3:**
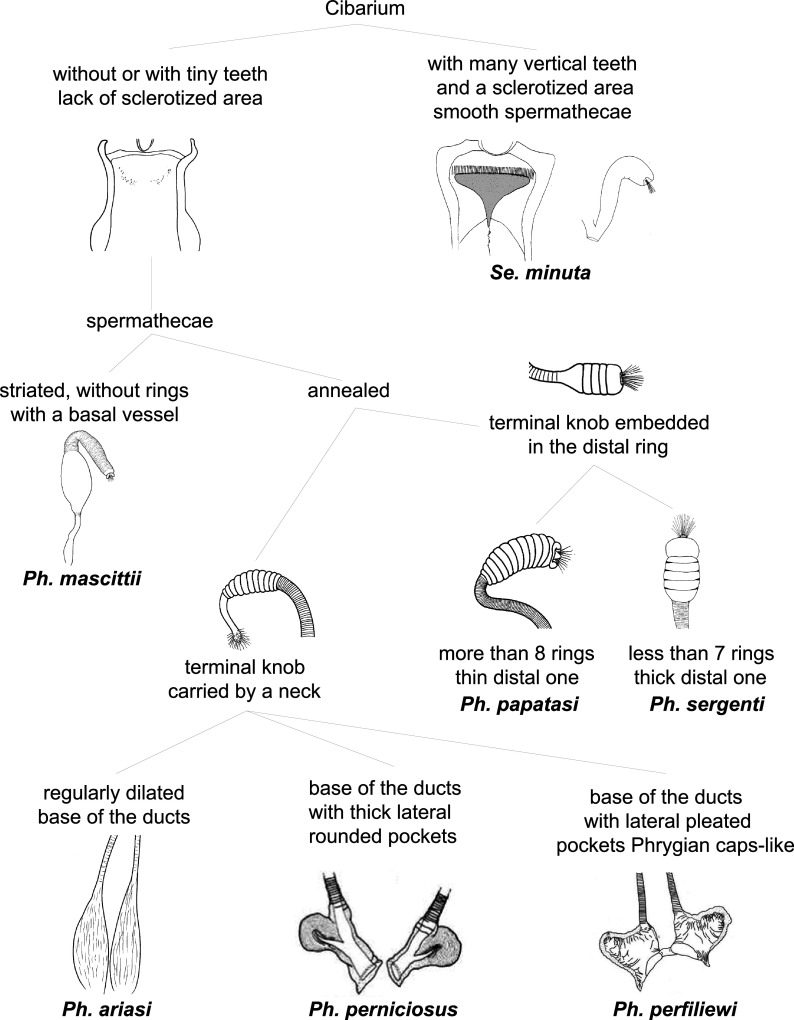
Simplified key for female sand fly species found in France.

**Figure 4:**
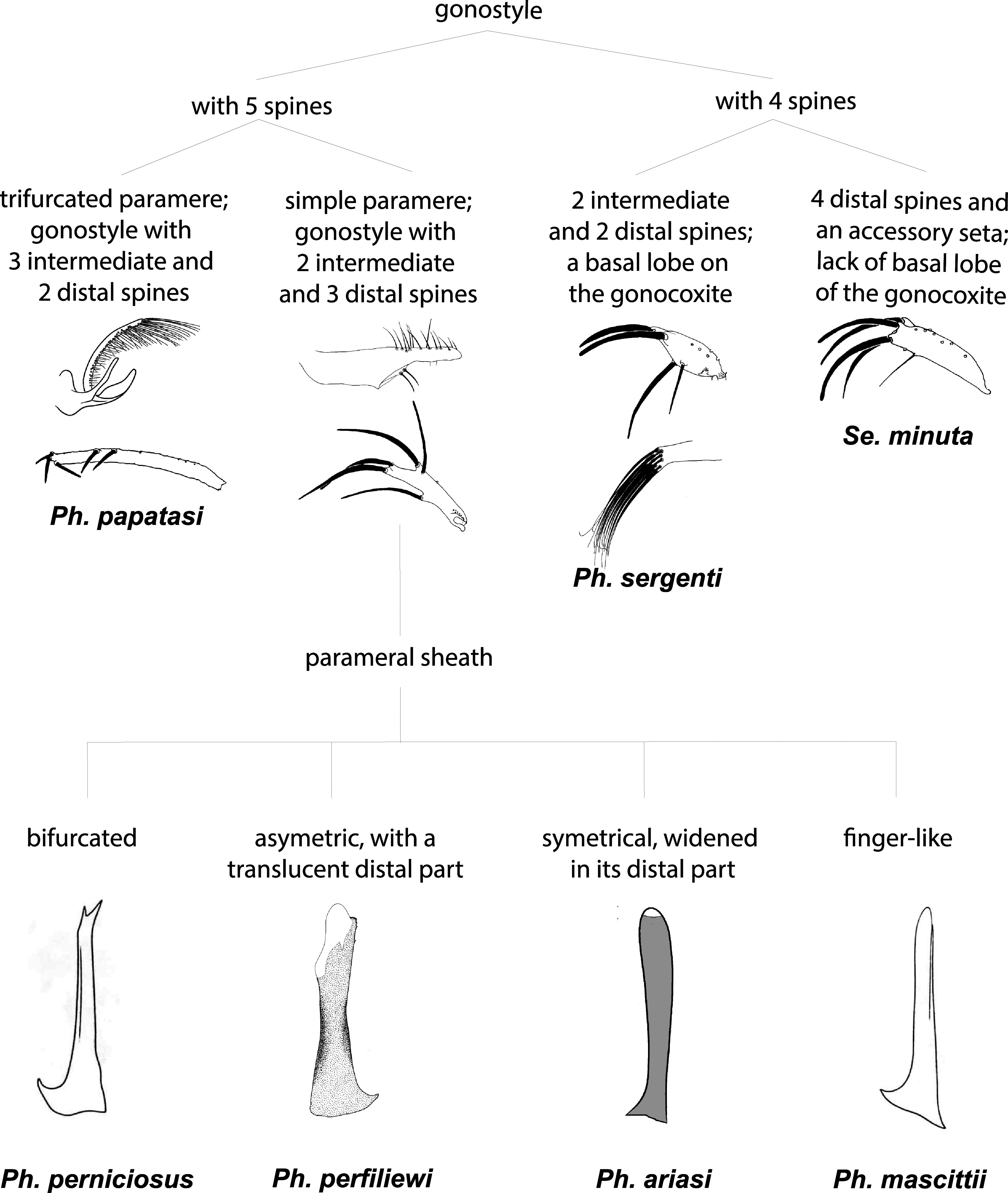
Simplified key for male sand fly species found in France.

In our systematic review, 7 species were recorded in France: *Ph. perniciosus*, *Ph. ariasi*, *Ph. papatasi*, *Ph. mascittii*, *Ph. perfiliewi*, *Ph. sergenti*, and *Se. minuta*. The number of studies reporting each species is summarized in Figure 2. In order to simplify the identification of species found in France for future studies, a simplified key for females is presented in Figure 3 and for males in Figure 4.

### Distribution of sand fly species

**Figure 5:**
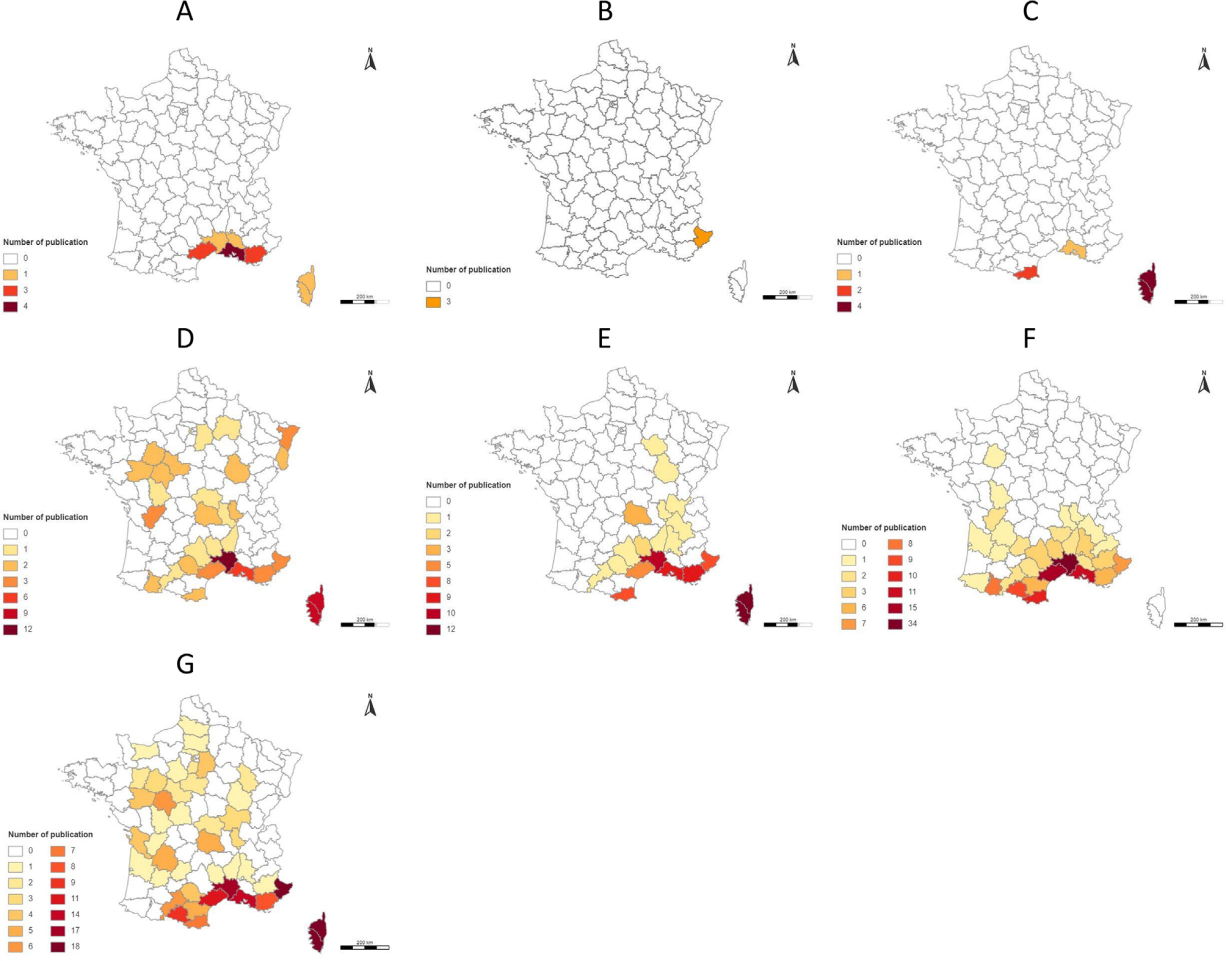
Distribution map by department according to the number of references between 1906 and 2023 for *Phlebotomus papatasi* (A), *Phlebotomus perfiliewi* (B), *Phlebotomus sergenti* (C), *Phlebotomus mascittii* (D), *Sergentomyia minuta* (E), *Phlebotomus ariasi* (F), and *Phlebotomus perniciosus* (G).

**Figure 6:**
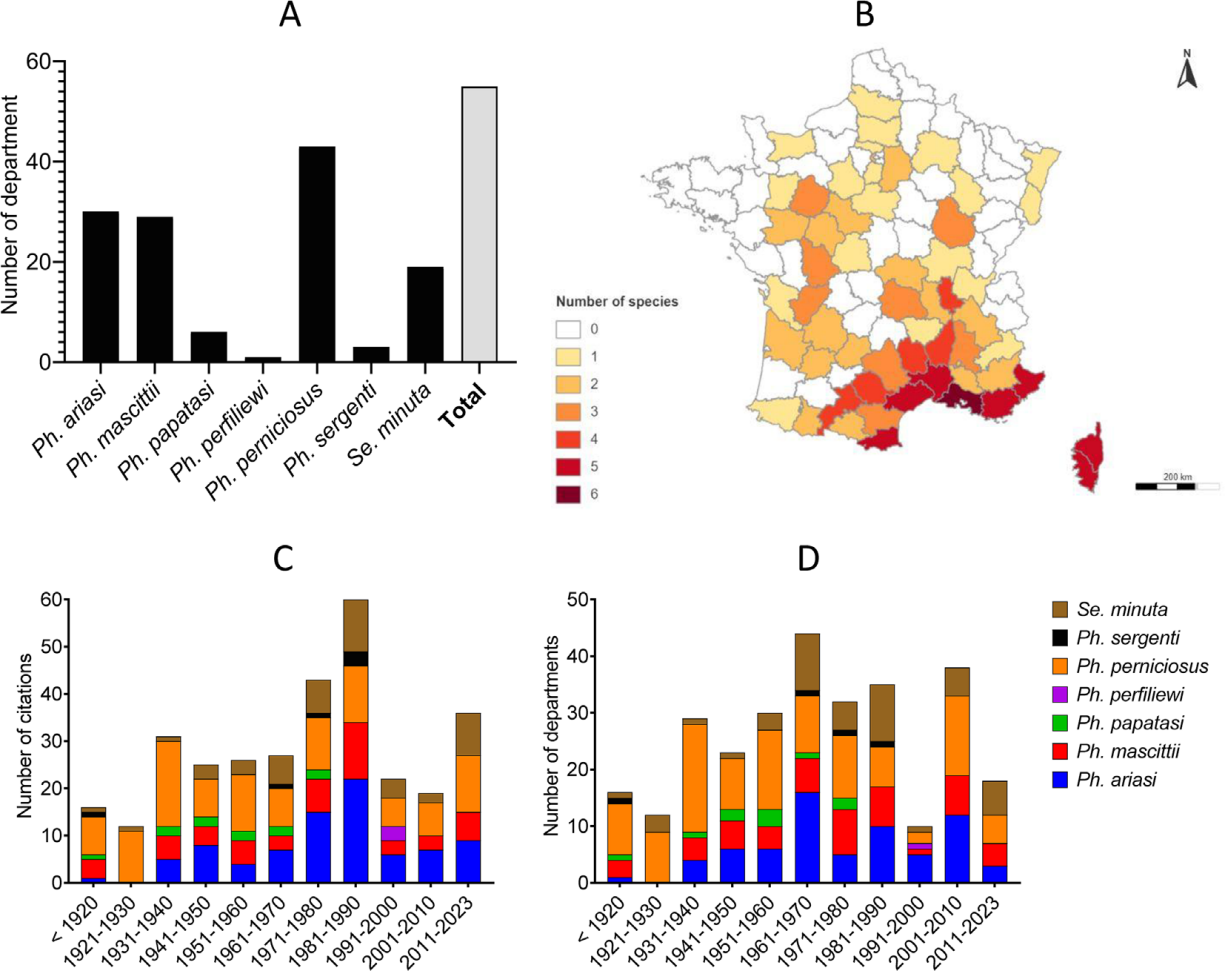
Number of recorded department by species (A), distribution map of the number of recorded species by department (B), number of recorded citations (C), and department (D) by species and by decade.

**Supplementary Figure 1:**
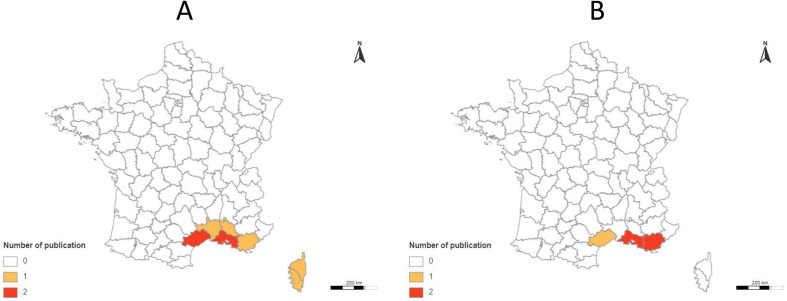
Distribution map of *Phlebotomus papatasi* by department according to the number of references between 1906 and 1956 (A) and between 1969 and 1977 (B).

**Supplementary Figure 2:**
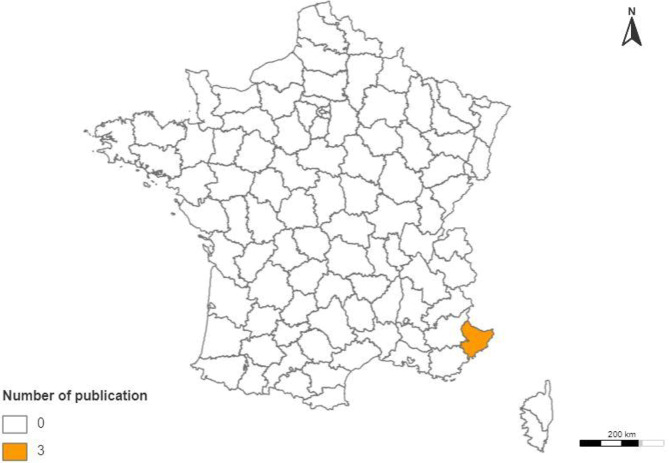
Distribution map of *Phlebotomus perfiliewi* by department according to the number of references between 1991 and 1993.

**Supplementary Figure 3:**
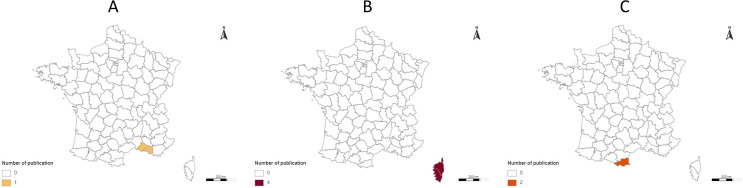
Distribution map of *Phlebotomus sergenti* by department according to the number of references in 1918 (A), 1969–1978 (B), and 1981–1984 (C).

**Supplementary Figure 4:**
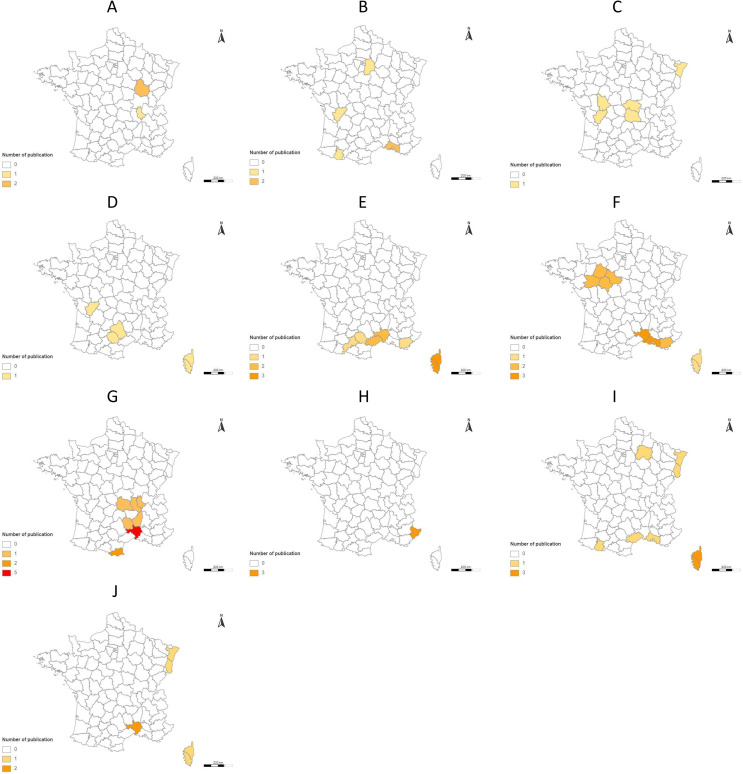
Distribution map of *Phlebotomus mascittii* by department according to the number of references before 1920 (A), 1931–1940 (B), 1941–1950 (C), 1951–1960 (D), 1961–1970 (E), 1971–1980 (F), 1981–1990 (G), 1991–2000 (H), 2001–2010 (I), and 2011–2023 (J).

**Supplementary Figure 5:**
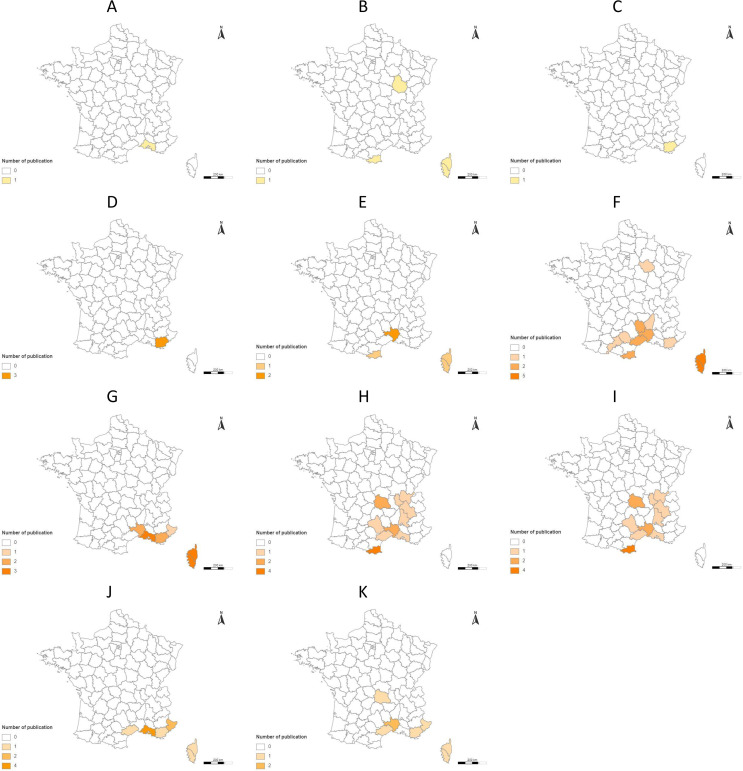
Distribution map of *Sergentomyia minuta* by department according to the number of references before 1920 (A), 1921–1930 (B), 1931–1940 (C), 1941–1950 (D), 1951–1960 (E), 1961–1970 (F), 1971–1980 (G), 1981–1990 (H), 1991–2000 (I), 2001–2010 (J), and 2011–2023 (K).

**Supplementary Figure 6:**
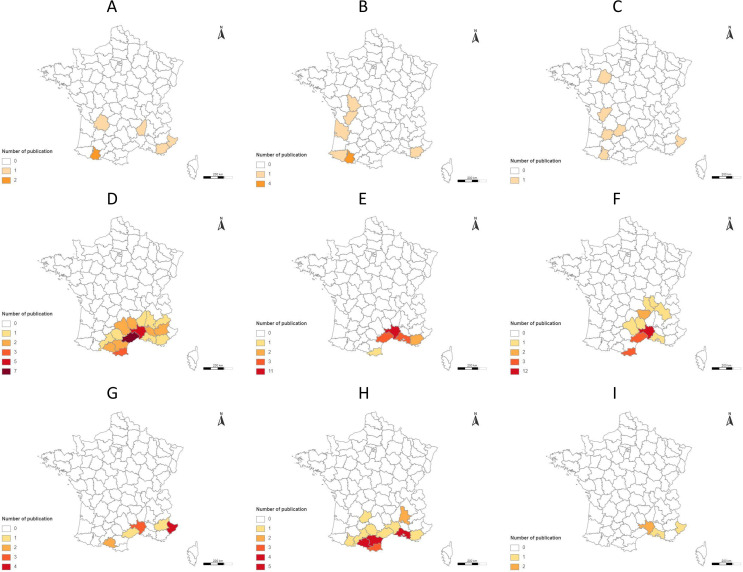
Distribution map of *Phlebotomus ariasi* by department according to the number of references between 1908 and 1940 (A), 1941–1950 (B), 1951–1960 (C), 1961–1970 (D), 1971–1980 (E), 1981–1990 (F), 1991–2000 (G), 2001–2010 (H), and 2011–2023 (I).

**Supplementary Figure 7:**
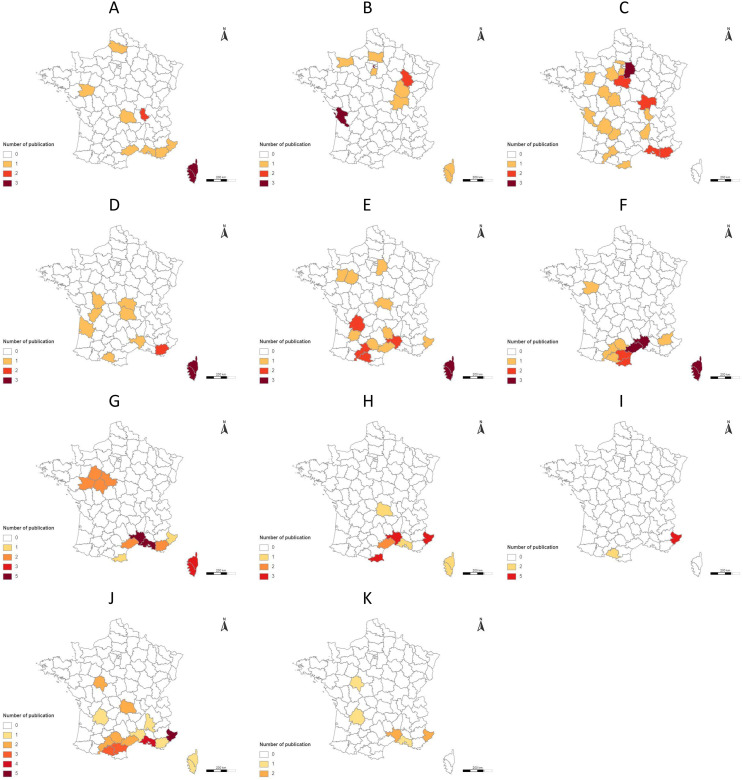
Distribution map of *Phlebotomus perniciosus* by department according to the number of references before 1920 (A), 1921–1930 (B), 1931–1940 (C), 1941–1950 (D), 1951–1960 (E), 1961–1970 (F), 1971–1980 (G), 1981–1990 (H), 1991–2000 (I), 2001–2010 (J), and 2011–2023 (K).

The cumulative data of studies recording the presence of the 7 sand fly species in France are available in Figure 5. *Phlebotomus papatasi*, *Ph. perfiliewi*, *Ph. sergenti*, *Ph. mascittii*, *Se. minuta*, *Ph. ariasi*, and *Ph. perniciosus* were recorded in 6, 1, 3, 29, 19, 30 and 43 departments, respectively (Fig. 6). All extracted information by species is summarized in Supplemental Table 2; all references are provided in Supplemental Table 1, and the detailed distribution maps for the different species by decade according to the number of references are available in Supplemental Figures 1–7. Finally, in order to visualize the data over time, we have also summarized the number of recorded citations and departments by species and decade (Fig. 6). A few corrections of species identification have been incorporated in this work for *Ph. papatasi*. Before 1932, this species was the most frequently cited in France, because at first it was thought that all sand flies captured belonged to this species [28]. Until that year, Montpellier (Hérault department) was the only locality in mainland France where this species was identified with certainty. Therefore, species identification as *Ph. papatasi* was corrected as *Ph. perniciosus* or *Ph. mascittii* according to the new identification performed in 1932 by Langeron and Nitzulescu [28]. Similarly, the names of some species were updated. Of note, *Ph. mascittii* and *Se. minuta* were designated as *Phlebotomus larroussei* until 1954, and *Phlebotomus parroti* until 1947 or *Phlebotomus minutus* until 1963, respectively. Therefore, the species identification was corrected accordingly in our systematic review.

### Abundance

**Figure 7:**
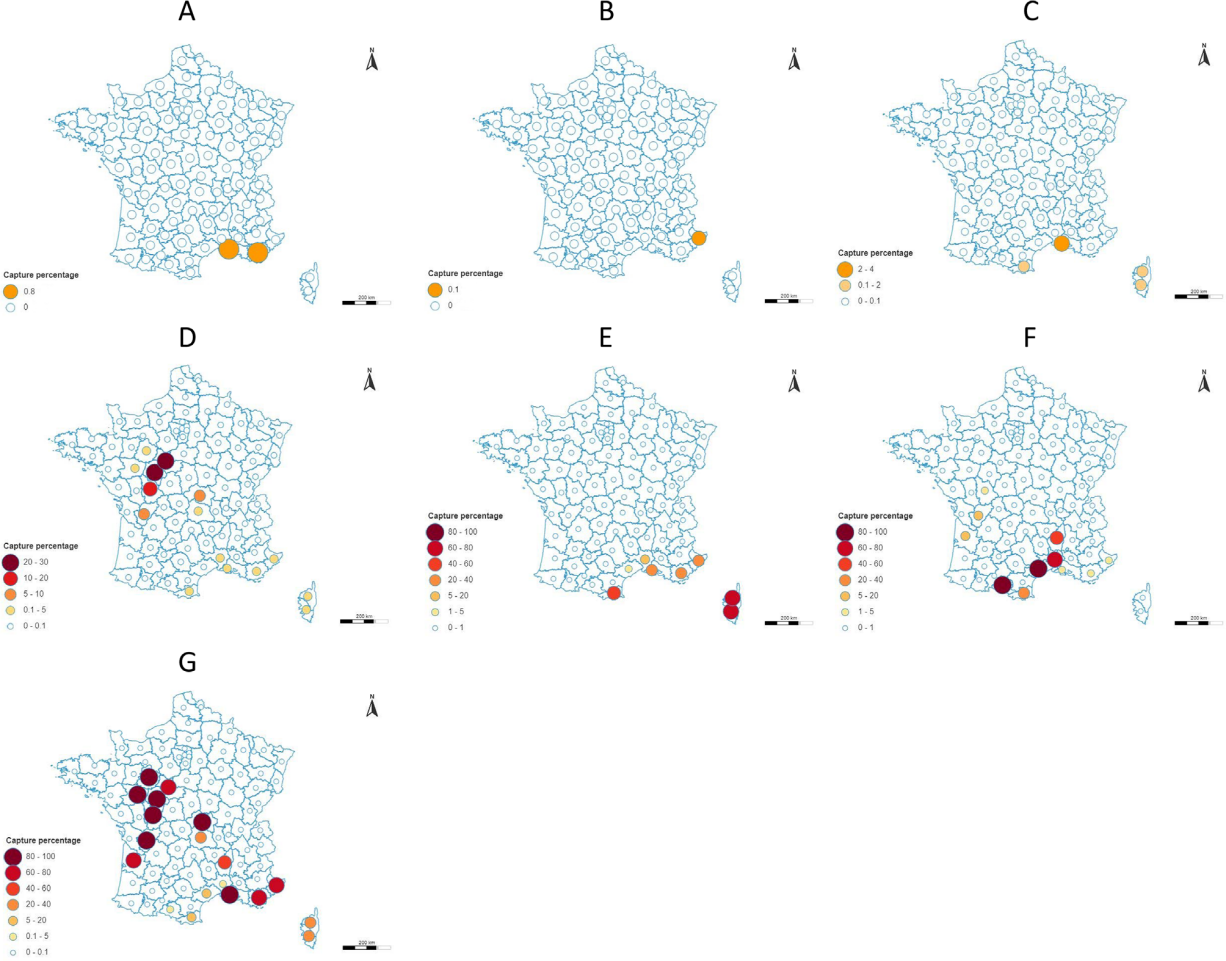
Distribution map of the different sand fly species by department according to the percentage of captures for *Phlebotomus papatasi* (A), *Phlebotomus perfiliewi* (B), *Phlebotomus sergenti* (C), *Phlebotomus mascittii* (D), *Sergentomyia minuta* (E), *Phlebotomus ariasi* (F), and *Phlebotomus perniciosus* (G).

Abundance, defined as the number of individuals captured or as a percentage of species captured by traps or by trapping period, was described in 81 reports (*n* = 81/172; 47.1%). However, these data are highly variable and localized. It is almost impossible to compare abundances, as studies do not have the same scientific questions or aims (e.g., presence record, infection rates studies, Mark-Recapture trials), and data collection (e.g., trapping method, abundance calculation in percentage or by m^2^) is not always comparable. There were very few studies that focused on the distribution and abundance of different species over time. However, in foci of transmission, abundance rates can provide good information (host-vector contact, trophic preferences, infection rate, etc.) on the structure of these foci and improve our understanding of transmission epidemiology. We have therefore summarized the body of data available in an abundance map by species (Fig. 7) and synthetized data in Table 2. These data take into account only the studies providing a species percentage by capture and should be considered qualitative data.

**Table 2:** Abundance, recent detection, vector competence, and expansion risk summarized by species.

Species	Abundance	Abundance range	Detection in recent reports	Vector competence	Risk of expansion
*Ph. papatasi*	Rare	1–34 ind	No	*Le. major*	No
*Ph. perfiliewi*	Rare	0.07–1%	No	*Le. infantum*	No
*Ph. sergenti*	Rare	0.1–3.6%	No	*Le. tropica*	To survey
*Ph. mascittii*	Rare	1%	Yes	Possible vector of *Leishmania* and Toscana virus	Yes
*Se. minuta*	Average or high	26–80%	Yes	Possible vector of Toscana virus	No
*Ph. ariasi*	Low, average, or high	1–2.7%	Yes	*Le. infantum*	No
		20–50%			
		85–98%			
*Ph. perniciosus*	From low to high	0.3–14%	Yes	*Le. infantum* Toscana virus	Yes
		74–98%			

## Discussion

### Bibliometric data

Based on the 172 documents collected, there are data gaps for many regions of France, making it impossible to determine the presence of sand flies in these areas. On average, 28 references were published per decade (Fig. 2). We can observe several peaks in publications (1930s, 1970s, and 1980s), which are the result of growing expertise associated with the description of new capture localities and, above all, numerous publications on the taxonomy of the various species in the 1930s and of field work carried out by Prof. Rioux and Prof. Killick-Kendrick and their teams in the other two decades. However, with an average of 3 publications by year, the number of publications remains low.

### Trapping practices

The first trappings were performed using manual capture (mouth aspirators). This technique was used until the 1980s, but was gradually replaced by sticky traps from the 1960s onwards. CDC Light traps have been used since the late 1970s. Other techniques (e.g., human bait capture, tunnel traps, or emergence traps) have also been deployed, but these represent only a few special cases. They are almost no longer used, either for ethical reasons (human bait capture) or for technical reasons (tunnel traps or emergence traps). Sticky traps and CDC Light traps have become the gold standard for sand fly captures [3]. Sticky traps can capture large numbers of individual over a wide area. They are interception traps that are inexpensive and easy to prepare and use in large numbers [3]. Light traps attract host-seeking sand flies. Their efficiency varies by gender, phototrophic behaviour, and species, but they are able to catch a sufficient proportions of individuals [21]. The trapping method is mostly determined by the objectives of the study, but also by the capacity of the team and the budget. Adapted capture techniques ensure representative sampling of insect populations, enabling valuable insights into insect ecology and population dynamics.

### Sand fly presence

Sand flies have been recorded in 55 departments. However, their absence in the other departments of France is certainly an artefact of the lack of data. Few data were published per decade, regardless of the species (Fig. 6). It is therefore almost certain that sand flies are present throughout France. However, we can observe a greater diversity of sand fly species in the Mediterranean region (Fig. 5), which is consistent with our knowledge of the ecology of the different species present in France.

Most of these documents do not provide climatic/environmental data, so it is not possible to determine the role of climate or rainfall on the distribution of sand fly species. However, in the current context of environmental change, an update of the presence of the various species in correlation with climatic data seems essential. As activity, development, and survival of sand flies are mostly influenced by temperature and humidity [52], the climate changes observed in recent years may greatly increase the risk of transmission [46].

Nevertheless, as the distributions of the various species is difficult to compare as a single entity due to the diversity of catching methods, the data are interpreted by species. We thus organized the species into three categories: (1) low abundance species, (2) abundant species that are little studied, and (3) abundant vector species.

### Low abundance species

***Phlebotomus papatasi*** is one of the most widespread and well-known *Phlebotomus* species (first description in 1786). It is very abundant in some geographic regions (e.g., Morocco and Algeria [26]), and it has a large size and is very aggressive towards humans [1]. This species is the main vector of *Leishmania major* [5]. The biology of this species is one of the best known, as it is relatively easy to breed. However, this species is only rarely found in France (Fig. 5) and has not been recorded since 1977 [45] (Fig. 2). Its presence in Corsica is questionable, as only a single individual was captured once in 1954. In departments where its presence is more likely (Var, Vaucluse, Bouches-du-Rhône, Hérault, and Gard), it was always caught in low abundance (Fig. 7). Despite the availability of more recent studies in these areas (e.g., Gard in 2011–2013 [44], or Bouches-du-Rhône in 2009–2011 [11, 15]), *Ph. papatasi* has not been captured again. However, the absence of captures should not be taken as proof of species disappearance, but rather as the absence of recent studies seeking this species. Taking into account, on the one hand, the scarcity of *Ph. papatasi*, but on the other, and above all, the absence of reservoir rodents, the risk of introduction and then endemization of *L. major* leishmaniasis does not yet exist in France.

***Phlebotomus perfiliewi*** is a zoophilic species with a wide distribution area around the Mediterranean basin [13, 48]. However, this species was rarely captured in France (Fig. 5). Captures were recorded only in the 1990s in the Alpes-Maritimes department [16, 24, 25] and always with a low abundance (Fig. 7). This species was not found in the most recent studies carried out in this department [10, 17, 22, 40]. Still, given its low abundance, it is difficult to state whether it is still present. A very low or discontinuous distribution may very well be an artefact due to the highly fragmentary information available.

***Phlebotomus sergenti*** prefers to bite warm-blooded animals, and is usually found in caves and houses in rural and urban areas, as well as in open landscape [1]. This anthropophilic species is a vector of *Leishmania tropica* [12]. It is extremely common in some countries (e.g., Morocco [14]), but in France, although captured as early as 1918 [41], it has rarely been found to date (Fig. 7). However, it is important to note that the most recent captures are more than 40 years old. Therefore, its presence in France may be debated given the lack of recent data. Monitoring the dynamics of this species is very important. Importantly, *L. tropica* leishmaniasis is an anthroponosis not needing a vertebrate reservoir. Therefore, the combination of leishmanial patients and relatively abundant populations of *Ph. sergenti* may in theory be sufficient for the endemization of this disease. The combined action of global change on the abundance of populations of this vector and the immigration of people from endemic areas could create the necessary determinants.

***Phlebotomus mascittii*** is known to be cavernicolous, but its biology is still poorly understood [34, 49]. However, studies attest its anthropophilic nature and its presence near foci of autochthonous cases of leishmaniasis in some regions of Europe [3, 18]. This species is always found in low population densities. In fact, out of all the documents containing information on the abundance of this species (*n* = 30), they accounted for at most 1% of captures (Fig. 7), with the rare exception of studies carried out in resting (or emergence) sites relatively specific to this species (e.g., more than 200 individuals captured in a tunnel in Corsica [34]). *Phlebotomus mascittii* is resistant to low temperatures, which explains its presence in northern Europe (e.g., Germany [33]). Although this species is increasingly studied, as it may play a role as a secondary vector for *Leishmania* and Phlebovirus [4, 36], we do not have much data. Even though this species was recorded in 29 departments (Fig. 6), this distribution likely extends to the neighboring departments, and likewise to the rest of France. Given its vector capacity, the monitoring of its distribution and abundance would be of interest to document its expansion and prevent outbreaks of vector-borne diseases.

### Abundant but little studied species

***Sergentomyia minuta*** is exophilic, almost exclusively zoophilic, preferring to feed on cold-blooded animals and bites humans only very exceptionally [20]. Although this species was recorded in only 19 departments (Fig. 6), it is certainly more widely distributed. The distribution data are partial, reflecting the few studies available in the literature, but not its actual presence. In the absence of its documented role as a vector, this species is still very little studied. However, it should not be neglected as its role is still debated, either as a vector (e.g., in Senegal [50]) or as a reservoir (e.g., Phlebovirus RNA found in individuals from the south of France [9]). Furthermore, this species can represent a significant percentage of the capture (Fig. 7).

### Abundant vector species

***Phlebotomus ariasi*** is ubiquitous and opportunistic. Even though the first captures of this species were recorded in 1908 in the Alpes-Maritimes department [6, 35], there seems to be no extension of the species further north of the Cevennes region. However, as seen in Figure 5, data are sparse. This species is mainly found in mixed oak forests and at altitudes ranging from 200 to 1400 m. In the Cevennes region, this species has a high density [42], whereas it is less abundant on the Mediterranean coastal plain [7, 47]. Indeed, it is highly abundant in the Pyrénées-Orientales, Gard, Ariège, and Hérault and at low densities in the other departments (Fig. 7). This species needs to be monitored more intensively given its important vector role in the areas where it is most abundant.

***Phlebotomus perniciosus*** is an opportunistic species and the principal vector of pathogens in France. This species is the most documented in France, certainly due to its role as a vector. In contrast to *Ph. ariasi*, *Ph. perniciosus* is the most abundant species in the Mediterranean-type vegetation [11] and can be found throughout southern France at altitudes below 600 m, with relative highest presence probability between 100 and 300 m [51]. Its abundance is opposite to that of *Ph. ariasi*. It is abundant in the Bouches-du-Rhône and Alpes-Maritimes, while it is found in low density in the Gard, Ariège, and Hérault (Fig. 7). As for *Ph. ariasi*, there seems to be no extension of the distribution area of *Ph. perniciosus*, with the limitation of the lack of a vector survey.

## Conclusion

Overall, it is important to note that these data are spotty and cannot be generalized to the whole country. Additionally, it is not possible to state whether species are increasing or decreasing, as there are no comparable datasets over time. These data highlight the need for a national, coordinated capture plan. This is the context of the European Climos project (Climate Monitoring and Decision Support Framework for Sand Fly-borne Diseases Detection and Mitigation, https://climos-project.eu/). The aim of this project is to better understand the climatic and environmental drivers of sand fly-borne diseases in order to provide risk assessments for a variety of stakeholders. The data resulting from this work, by providing interactive mapping and information services accessible to the public, will be used to update distribution and abundance data for these sand fly species.

## Data Availability

All resources used in this article are provided in the Supporting Information and all the analyses are detailed allowing the assessment or verification of the manuscript’s findings.

## Supplementary material

The supplementary material for this article can be found at https://www.parasite-journal.org/10.1051/parasite/2024045/olm.

*Supplementary Table 1*: Complete list of references included in the systematic review. *Supplementary Table 2*: Captures of sand fly species in France between 1906 and 2023 (NA: data not provided).
