# Supplementary material for: Phlebotomine sand fly distribution and abundance in France: A systematic review
Source: Parasite. 2024 Aug 7;31:45. doi: 10.1051/parasite/2024045 (PMC11305120; doi:10.1051/parasite/2024045)
Supplement: Supplementary file 1 — Supplementary Table 1: Complete list of references included in the systematic review. Supplementary Table 2: Captures of sand fly species in France between 1906 and 2023 (NA: data not provided). [file parasite-31-45-s1.zip › parasite240087-1-olm/Supplementary_Table_1.pdf]

Supplementary Table 1. Complete list of references included in the systematic review.

1. Abonnenc E, Poinsot S, Rioux J-A. 1971. Tératologie des phlébotomes (Diptera: Psychodidae). Révision et nouvelles observations. Cahier de l'ORSTOM, série Entomologie Médicale et Parasitologie, IX, 307-316.
2. Aubert P. 1912. Le *Phlebotomus papatacci* dans la région lyonnaise. Bulletin du Lyon Médical, 2, 580-583.
3. Ballart C, Pesson B, Gallego M. 2018. Isoenzymatic characterization of *Phlebotomus ariasi* and *P. perniciosus* of canine leishmaniasis foci from Eastern Pyrenean regions and comparison with other populations from Europe. Parasite, 25, 3.
4. Baudrimont A. 1943. Présence de *Phlebotomus ariasi* Tonnoir, 1921 à Saint-Sauveur (Hautes-Pyrénées). Actes de la Société Linéenne de Bordeaux, 93, 46-58.
5. Baudrimont A. 1946. Nouvelles observations sur la présence de *Phlebotomus ariasi* Tonnoir, 1921 à Saint-Sauveur (Hautes-Pyrénées). Actes de la Société Linéenne de Bordeaux, 96, 204-207.
6. Baudrimont A. 1947. Brunissement progressif des femelles de *Phlebotomus ariasi* Tonnoir, 1921 au cours de la dernière étape de leur évolution sexuelle. Actes de la Société Linéenne de Bordeaux, 6, 27-30.
7. Baudrimont A. 1955. Quelques observations et réflexions sur la biologie de *Phlebotomus ariasi* Tonnoir, 1921, à Saint-Sauveur (Hautes-Pyrénées) au cours des années 1950-1951-1952-1953-1954. Procès-Verbaux de la Société Linnéenne de Bordeaux, 96, 28-30.
8. Beati L. 1989. La leishmaniose en Corse, Résultats d'une enquête éco-épidémiologique. Thesis. Faculté de Médecine: Lausanne (Switzerland).
9. Benabdennbi I. 1998. Contribution à l'étude de la biodiversité chez les *Larroussius* (Diptera: Psychodidae) du bassin méditerranéen. Apports de l'analyse isoenzymatique. Thesis. Université Louis Pasteur: Strasbourg (France).
10. Benabdennbi I, Pesson B. 1998. A propos de la variabilité morphologique de *Phlebotomus perniciosus* (Diptera : Psychodidae). Bulletin de la Société Française de Parasitologie, 16(1), 53-60.
11. Benlarbi M, Ready PD. 2003. Host-specific *Wolbachia* strains in widespread populations of *Phlebotomus perniciosus* and *P. papatasi* (Diptera: Psychodidae), and prospects for driving genes into these vectors of *Leishmania*. Bulletin of Entomological Research, 93(5), 383-391.
12. Bichaud L, Izri A, de Lamballerie X, Moureau G, Charrel RN. 2014. First detection of Toscana virus in Corsica, France. Clinical Microbiology and Infection, 20(2), O101-104.
13. Blanchard R. 1909. À propos des *Phlebotomus* [Dipt. Psychodidae]. Bulletin de la Société Entomologique de France, 14(11), 192-195.
14. Bourgain M. 1945. Contribution à l'étude des phlébotomes du littoral méditerranéen français. Présence de *Phlebotomus* (*Brumptius*) *parroti*. Adler et Theodor 1926 aux Darboussèdes (Banlieue de Toulon). Bulletin de la Société de Pathologie Exotique, 38, 150-158.
15. Bourgain M. 1945. Sur un exemplaire de *Phlebotomus papatasi* Scopoli, 1786, capturé à Toulon. Bulletin de la Société de Pathologie Exotique, 38, 145-149.
16. Bourgain M. 1949. Présence de *Phlebotomus* (*Larroussius*) *ariasi* Tonnoir, 1921 dans les Basses-Pyrénées. Bulletin de la Société de Pathologie Exotique, 42, 562-563.
17. Bourgain M. 1949. Présence de *Phlebotomus* (*Larroussius*) *ariasi* Tonnoir, 1921 et de *Phlebotomus* (*Larroussius*) *perniciosus* Newstead, 1911 en Gironde Bulletin de la Société de Pathologie Exotique, 42, 564-565.
18. Bourgain M. 1958. Présence de *Phlebotomus* (*Larroussius*) *perniciosus* Newstead, 1911 en Lozère. Bulletin de la Société de Pathologie Exotique, 51, 534-535.
19. Boussaa S, Boumezzough A, Sibold B, Alves-Pires C, Marquez FM, Glasser N, Pesson B. 2009. Phlebotomine sandflies (Diptera: Psychodidae) of the genus *Sergentomyia* in Marrakech region, Morocco. Parasitology Research, 104, 1027-1033.
20. Callot J. 1944. Localités nouvelles pour quelques Nématocères piqueurs. Annales de Parasitologie Humaine et Comparée, XX(3-4), 207-208.
21. Callot J. 1950. Notes et informations: Présence de *Phlebotomus larroussei* en Alsace. Annales de Parasitologie Humaine et Comparée, 25(1-2), 112.
22. Chambost L, Houdemer E. 1947. Capture de *Phlebotomus perniciosus*, Newstead 1911, à Ajaccio (Corse). Bulletin de la Société de Pathologie Exotique, 40, 361-362.
23. Charrel RN, Izri A, Temmam S, de Lamballerie X, Parola P. 2006. Toscana virus RNA in *Sergentomyia minuta* flies. Emerging Infectious Diseases, 12(8), 1299-1300.
24. Charrel RN, Izri A, Temmam S, Delaunay P, Toga I, Dumon H, Marty P, de Lamballerie X, Parola P. 2007. Cocirculation of 2 genotypes of Toscana virus, southeastern France. Emerging Infectious Diseases, 13(3), 465-468.
25. Clastrier J. 1938. Observations sur les phlébotomes de la région de Privas (Ardèche). Archives de l'Institut Pasteur d'Algérie, 16(1), 31-35.
26. Colas-Belcour J. 1939. Présence de *Phlebotomus perniciosus* en Seine-et-Marne. Bulletin de la Société de Pathologie Exotique, 32, 901.
27. Colas-Belcour J. 1956. Sur la présence de phlébotomes en Allier. Bulletin de la Société de Pathologie Exotique, 49(6), 1116-1120.
28. Colas-Belcour J. 1957. Phlébotomes et Leishmanioses autochtones en France. Archives de l'Institut Pasteur du Maroc, 5(6), 243-261.
29. Colas-Belcour J, Abonnenc E. 1948. Contribution à l'étude de *Phlebotomus* (*Prophlebotomus*) *minutus* Rondani en France. Bulletin de la Société de Pathologie Exotique, 41, 229-232.
30. Colas-Belcour J, Tisseuil J. 1936. Nouvelle contribution à la répartition des phlébotomes en France. Revue de Pathologie Comparée et Hygiène Générale, 36, 117-123.
31. Colas-Belcour MC, Colas-Belcour J. 1929. Présence de *Phlebotomus perniciosus* Newstead dans le département du Calvados. Bulletin de la Société Linnéenne de Normandie, 2, 4-6.

32. Cotteaux-Lautard C, Leparc-Goffart I, Berenger JM, Plumet S, Pages F. 2016. Phenology and host preferences *Phlebotomus perniciosus* (Diptera: Phlebotominae) in a focus of Toscana virus (TOSV) in South of France. Acta Tropica, 153, 64-69.
33. Coudert J. 1947. Présence de *Phlebotomus papatasi* (Scopoli 1786) à Gigondas (Vaucluse). Publications de la Société Linnéenne de Lyon, 16(3), 53-54.
34. Croset H, Rioux J, Leger N, Houin R, Soussi M, Benmansour N, Maistre M. 1969. Les méthodes d'échantillonnage des populations de phlébotomes en région méditerranéenne, in: Ecologie des leishmanioses. Colloques lnternationaux du CNRS. CNRS: Paris (France). p. 139-151.
35. Depaquit J, Hadj-Henni L, Bounamous A, Strutz S, Boussaa S, Morillas-Marquez F, Pesson B, Gallego M, Delecolle JC, Afonso MO, Alves-Pires C, Capela RA, Couloux A, Leger N. 2015. Mitochondrial DNA intraspecific variability in *Sergentomyia minuta* (Diptera: Psychodidae). Journal of Medical Entomology, 52(5), 819-828.
36. Depaquit J, Naucke TJ, Schmitt C, Ferté H, Léger N. 2005. A molecular analysis of the subgenus *Transphlebotomus* Artemiev, 1984 (Phlebotomus, Diptera, Psychodidae) inferred from ND4 mtDNA with new northern records of *Phlebotomus mascittii* Grassi, 1908. Parasitology Research, 95(2), 113-116.
37. Dereure J, Vanwambeke SO, Male P, Martinez S, Pratlong F, Balard Y, Dedet JP. 2009. The potential effects of global warming on changes in canine leishmaniasis in a focus outside the classical area of the disease in southern France. Vector-Borne and Zoonotic Diseases, 9(6), 687-694.
38. Durand-Delacre R. 1949. Sur quelques phlébotomes de la Charente. Archives de l'Institut Pasteur d'Algérie, 27(1), 39-41.
39. Dye C, Guy MW, Elkins DB, Wilkes TJ, Killick-Kendrick R. 1987. The life expectancy of phlebotomine sandflies: first field estimates from southern France. Medical and Veterinary Entomology, 1(4), 417-425.
40. Erisoz Kasap O, Dvorak V, Depaquit J, Alten B, Votypka J, Volf P. 2015. Paleobiogeography of the subgenus *Transphlebotomus* Artemiev with description of two new species, *Phlebotomus anatolicus* n. sp. and *Phlebotomus killicki* n. sp. . Infection, Genetics and Evolution, 34, 467-479.
41. Esseghir S, Ready PD, Killick-Kendrick R, Ben-Ismail R. 1997. Mitochondrial haplotypes and phylogeography of *Phlebotomus* vectors of *Leishmania major*. Insect Molecular Biology, 6(3), 211-225.
42. Faucher B, Bichaud L, Charrel R, Mary C, Izri A, de Lamballerie X, Piarroux R. 2014. Presence of sandflies infected with *Leishmania infantum* and Massilia virus in the Marseille urban area. Clinical Microbiology and Infection, 20(5), O340-343.
43. Fauran P, Izri A, Delaunay P, Marty P. 1998. Les phlébotomes (Diptera, Phlebotominae) des Alpes-Maritimes et de Monaco. Riviéra Scientifique, 82, 41-48.
44. Foley H. 1923. Présence de *Phlebotomus perniciosus* Newstead dans le département de la Haute-Marne. Bulletin de la Société de Pathologie Exotique, 16, 664.
45. Foley H. 1924. Existence de *Phlebotomus perniciosus* Newstead à Vignory (Haute-Marne). Bulletin de la Société de Sciences Naturelles de la Haute-Marne, 7(1), 247-248.
46. Franca C, Roubaud E. 1921. Sur la détermination spécifique d'une femelle de phlébotome. Bulletin de la Société de Pathologie Exotique, 14, 23-24.
47. Franco FA, Morillas-Marquez F, Baron SD, Morales-Yuste M, Galvez R, Diaz V, Pesson B, Alves-Pires C, Depaquit J, Molina R, Afonso MO, Gallego M, Guernaoui S, Bounamous A, Martin-Sanchez J. 2010. Genetic structure of *Phlebotomus* (*Larroussius*) *ariasi* populations, the vector of *Leishmania infantum* in the western Mediterranean: epidemiological implications. International Journal for Parasitology, 40(11), 1335-1346.
48. GBIF. Global Biodiversity Information Facility. 2023 [cited 20/02/2023]; Available from: https://www.gbif.org/fr/.
49. Giacomo A. 1978. Épidémiologie de la leishmaniose canine dans les Alpes-Maritimes. Thesis. Ecole Nationale Vétérinaire d'Alfort: Maisons-Alfort (France).
50. Gibb PA, Anderson TJ, Dye C. 1988. Are nulliparous sandflies light-shy? Transactions of the Royal Society of Tropical Medicine and Hygiene, 82(2), 342-343.
51. Gilot B, Gillet JF, Quilici M, Dunan S, Lachet B, Ranque J. 1983. Le foyer Marseillais de leishmaniose viscérale : cartographie épidémiologique à 1/50 000. Essai méthodologique. Documents de Cartographie Ecologique, 26, 3-27.
52. Golvan YJ, Rioux JA, Chabaud AG. 1963. Infestation spontanée de phlébotomes par le spiruride *Mastophorus muris* (Gmelin). Annales de Parasitologie Humaine et Comparée, 38, 934.
53. Guilvard E, Rioux J-A, Jarry D, Moreno G. 1985. Accouplements successifs chez *Phlebotomus ariasi* Tonnoir, 1921. Annales de Parasitologie Humaine et Comparée, 60(4), 503-504.
54. Guilvard E, Wilkes TJ, Killick-Kendrick R, Rioux J. 1980. Écologie des leishmanioses dans le sud de la France. 15. Déroulement des cycles gonotrophiques chez *Phlebotomus ariasi* Tonnoir, 1921 et *Phlebotomus mascittii* Grassi, 1908 en Cévennes. Corollaire épidémiologique. Annales de Parasitologie Humaine et Comparée, 55(6), 659-664.
55. Guy MW, Killick-Kendrick R, Gill GS, Rioux JA, Bray RS. 1984. Ecology of leishmaniasis in the south of France. 19. Determination of the hosts of *Phlebotomus ariasi* Tonnoir, 1921 in the Cevennes by bloodmeal analyses. Annales de Parasitologie Humaine et Comparée, 59(5), 449-458.
56. Harant H, Rioux J-A. 1956. Considérations épidémiologiques sur les leishmanioses en Languedoc méditerranéen. Minerva medica, 8, 1-15.
57. Hartemink N, Vanwambeke S, Heesterbeek H, Rogers D, Morley D, Lambin E, Pesson B, Davies C, Mahamdallie S, Ready P. 2009. Modelling and mapping the basic reproduction number R0 for canine leishmaniasis: a case study for a region in South West France, in: Vector-borne diseases: the basic reproduction number R0 and risk maps. p. 111-138.
58. Henia A. 1983. Étude des phlébotomes (Diptera psychodidae) de la moyenne vallée du Rhône en 1982. Thesis. Ecole Nationale Vétérinaire de Lyon: Lyon (France).

59. Hide M, Marion E, Pomares C, Fisa R, Marty P, Bañuls A-L. 2013. Parasitic genotypes appear to differ in leishmaniasis patients compared with asymptomatic related carriers. International Journal for Parasitology, 43(5), 389-397.
60. Houin R. 1963. Données épidémiologiques et déductions prophylactiques sur les leishmanioses autochtones en France. Annales de Parasitologie Humaine et Comparée, 38(3), 379-438.
61. Houin R, Beaucournu J-C. 1966. Nouvelles stations françaises de Phlébotomes. Annales de Parasitologie Humaine et Comparée, 41(4), 387-390.
62. Houin R, Deniau M, Plel F, Reynouard F, Barbier D, Bonnet M, Plouton A, Pontegnie L. 1975. Phlébotomes de Touraine. Annales de Parasitologie Humaine et Comparée, 50(2), 233-243.
63. Houin R, Jolivet G, Combescot C, Deniau M, Puel F, Barbier D, Romano P, Kerboeuf D. 1977. Etude préliminaire d'un foyer de leishmaniose canine dans la région de Tours, in: Ecologie des leishmanioses. Colloques lnternationaux du CNRS. CNRS: Paris (France). p. 109-115.
64. Huguenin A, Pesson B, Kaltenbach ML, Diarra AZ, Parola P, Depaquit J, Randrianambinintsoa FJ. 2022. MALDI-TOF MS limits for the identification of Mediterranean sandflies of the subgenus *Larroussius*, with a special focus on the *Phlebotomus perniciosus* complex. Microorganisms, 10(11), 2135-2149.
65. INPN. Inventaire National du Patrimoine Naturel. Synthèse de données pour les espèces. 2023 [cited 20/02/2023]; Available from: https://inpn.mnhn.fr/espece/indicateur/FR/ES/7/CL/PH/Arthropoda.
66. Izri MA, Marty P, Fauran P, Le Fichoux Y, Rousset J-J. 1994. *Phlebotomus perfiliewi* Parrot, 1930 (Diptera, Psychodidae) dans le sud-est de la France. Parasite, 1(3), 286-286.
67. Izri MA, Marty P, Fauran P, Le Fichoux Y, Rousset JJ. 1996. Presumed vectors of leishmaniasis in the principality of Monaco. Transactions of the Royal Society of Tropical Medicine and Hygiene, 90(2), 114.
68. Izri MA, Marty P, Rahal A, Lelievre A, Ozon C, Baldelli G, Presiozo J, Haas P, Le Fichoux Y. 1992. *Phlebotomus perniciosus* Newstead, 1911 naturally infected by promastigotes in the region of Nice (France). Bulletin de la Société de Pathologie Exotique, 85(5), 385-387.
69. Jarry D. 1956. A propos d'un Phlébotome du groupe *minutus* capturé à Banyuls-sur-Mer. Vie et Milieu, 7(1), 114-115.
70. Kamhawi S, Molyneux DH, Killick-Kendrick R, Milligan PJ, Phillips A, Wilkes TJ, Killick-Kendrick M. 1987. Two populations of *Phlebotomus ariasi* in the Cevennes focus of leishmaniasis in the south of France revealed by analysis of cuticular hydrocarbons. Medical and Veterinary Entomology, 1(1), 97-102.
71. Killick-Kendrick R. 1987. Breeding places of *Phlebotomus ariasi* in the cevennes focus of leishmaniasis in the south of France. Parassitologia, 29(2-3), 181-191.
72. Killick-Kendrick R, Killick-Kendrick M. 1987. Honeydew of aphids as a source of sugar for *Phlebotomus ariasi*. Medical and Veterinary Entomology, 1, 297-302.
73. Killick-Kendrick R, Molyneux DH, Rioux JA, Lanotte G, Leaney AJ. 1980. Possible origins of *Leishmania chagasi*. Annals of Tropical Medicine & Parasitology, 74(5), 563-565.
74. Killick-Kendrick R, Rioux J-A, Ratify M, Guy MW, Wilkes TJ, Guy FM, Davidson I, Knechtli R, Ward RD, Guilvard E. 1984. Ecology of leishmaniasis in the south of France. 20. Dispersal of *Phlebotomus ariasi* Tonnoir, 1921 as a factor in the spread of visceral leishmaniasis in the Cévennes. Annales de Parasitologie Humaine et Comparée, 59(6), 555-572.
75. Killick-Kendrick R, Wilkes T, Alexander J, Bray RS, Rioux JA, Bailly M. 1985. The distance of attraction of CDC light traps to phlebotomine sandflies. Annales de Parasitologie Humaine et Comparée, 60(6), 763-767.
76. Killick-Kendrick R, Wilkes TJ, Bailly M, Bailly I, Righton LA. 1986. Preliminary field observations on the flight speed of a phlebotomine sandfly. Transactions of the Royal Society of Tropical Medicine and Hygiene, 80(1), 138-142.
77. Langeron M. 1912. Localités nouvelles de Phlébotomes. Comptes Rendus des Séances de la Société de Biologie et de ses Filiales, 72(1), 973-974.
78. Langeron M. 1916. Les Phlébotomes dans la région parisienne. Bulletin de la Société de Pathologie Exotique, 9, 573-576.
79. Langeron M. 1925. Phlébotomes de la région parisienne. Annales de Parasitologie Humaine et Comparée, 3(1), 104.
80. Langeron M, Nitzulescu V. 1932. Révision des phlébotomes de France. Annales de Parasitologie Humaine et Comparée, 10, 286-294.
81. Lanotte G, Rioux JA, Maazoun R, Pasteur N, Pratlong F, Lepart J. 1981. Application de la méthode numérique à la toxonomie du genre *Leishmania* Ross, 1903. A propos de 146 souches originaires de l'Ancien Monde. Utilisation des allozymes. Corollaires épidémiologiques et phylétiques. Annales de Parasitologie Humaine et Comparée, 56, 575-592.
82. Larrousse F. 1923. Présence de *Phlebotomus perniciosus* Newstead dans le département de l'Oise. Bulletin de la Société de Pathologie Exotique, 16, 16-17.
83. Larrousse F. 1925. Phlébotomes observés dans de nouvelles localités françaises. Annales de Parasitologie Humaine et Comparée, 3(1), 103.
84. Lavier G. 1935. Nouvelle localité française pour *Phlebotomus perniciosus*. Annales de Parasitologie Humaine et Comparée, 13(3), 279.
85. Lavier G. 1937. Localité française nouvelle pour *Phlebotomus perniciosus*. Annales de Parasitologie Humaine et Comparée, 15(1), 91.
86. Lavier G, Ristorcelli A. 1939. Localités nouvelles de Seine-et-Oise pour *Phlebotomus perniciosus*. Annales de Parasitologie Humaine et Comparée, 17(6), 592.
87. Lavier G, Ristorcelli A. 1939. Présence en Seine-et-Marne de *Phlebotomus larroussei*. Annales de Parasitologie Humaine et Comparée, 17(5), 375-379.
88. Le Chuiton F, Le Gac P, Penneac'h J. 1937. Présence à Toulon-sur-mer de *Phlebotomus perniciosus* Newstead 1911. Bulletin de la Société de Pathologie Exotique, 30, 698-699.
89. Le Gac P. 1936. Note sur la présence à Saint-Raphaël (Var) de *Phlebotomus perniciosus* Newstead, 1911. Bulletin de la Société de Pathologie Exotique, 29, 966.

90. Le Gac P. 1936. Sur trois phlébotomes capturés à Provins (Seine-et-Marne) pouvant être rapportés à *Phlebotomus perniciosus* Newstead 1911. Bulletin de la Société de Pathologie Exotique, 29(9), 966-970.
91. Le Gac P, Floch H, Chassignet R. 1952. Présence de *Phlebotomus* (*Larroussius*) *perniciosus* Newstead, 1911, et de *Phlebotomus* (*Larroussius*) *ariasi* Tonnoir, 1921, à La Théoule (Alpes-Maritimes). Bulletin de la Société de Pathologie Exotique, 45, 618-619.
92. Legendre J. 1916. Sur l'existence dans la Somme de *Phlebotomus papatasi* Scopoli. Comptes Rendus des Séances de la Société de Biologie et de ses Filiales, 79, 25-26.
93. Legendre J. 1924. Sur l'existence de *Phlebotomus perniciosus* en Saintonge. Bulletin de la Société de Pathologie Exotique, 17, 894-896.
94. Legendre J. 1929. Zoophily of *Phlebotomus* in Saintonge. Bulletin de l'Académie Nationale de Médecine, 102(34), 238-240.
95. Leger M, Seguinaud J. 1912. Fièvre de pappataci en Corse. Bulletin de la Société de Pathologie Exotique, 5, 710-714.
96. Leger N, Pesson B, Madulo-Leblond G. 1985. Nouvelles localisations en France de *Phlebotomus ariasi*, *P. mascittii* et *Sergentomyia minuta* (Diptera-Phlebotomidae). Annales de Parasitologie Humaine et Comparée, 60(3), 367-368.
97. Léger N, Pesson B, Madulo-Leblond G, Abonnenc E. 1983. Sur la différenciation des femelles du sous-genre *Larroussius* Nitzulescu, 1931 (Diptera-Phlebotomidae) de la région méditerranéenne. Annales de Parasitologie Humaine et Comparée, 58(6), 611-623.
98. Lesne P. 1909. Capture du *Phlebotomus pappataci* Scop. en Bourgogne (Dipt. Psychodidae). Bulletin de la Société Entomologique de France, 19, 333.
99. Lesne P. 1912. Captures du *Phlebotomus papatasi* Scop. en Bourgogne (2e note) (Dipt. Psychodidae). Bulletin de la Société Entomologique de France, 20, 410-411.
100. Mahamdallie SS. 2010. Effects of environmental change on the genetic diversity and distribution of *Phlebotomus ariasi*, a vector of visceral leishmaniasis in Southwest Europe. Thesis. London School of Hygiene & Tropical Medicine: London (England).
101. Mahamdallie SS, Pesson B, Ready PD. 2011. Multiple genetic divergences and population expansions of a Mediterranean sandfly, *Phlebotomus ariasi*, in Europe during the Pleistocene glacial cycles. Heredity, 106(5), 714-726.
102. Malé P. 2001. Étude éco-épidémiologique d'un foyer de leishmaniose à Tarascon sur Ariège. Thesis. Ecole Nationale Vétérinaire de Lyon: Lyon (France).
103. Mansion J. 1913. Diptères de Corse. Un nouveau moucheron vulnérant. Bulletin de la Société d'Histoire Naturelle de Corse, 123-144.
104. Mansion J. 1913. Les phlébotomes en Corse. Bulletin de la Société de Pathologie Exotique, 6(9), 637-641.
105. Mansion J. 1920. Une nouvelle station de phlébotomes en France. Bulletin de la Société de Pathologie Exotique, 13, 735-738.
106. Mirouse R. 1959. Diptères Psychodidés des Pyrénées ariégeoises. Bulletin de la Société d'Histoire Naturelle de Toulouse, 94, 325-330.
107. Naucke TJ, Menn B, Massberg D, Lorentz S. 2008. Winter activity of *Phlebotomus* (*Transphlebotomus*) *mascittii*, Grassi 1908 (Diptera: Psychodidae) on the island of Corsica. Parasitology Research, 103(2), 477-479.
108. Nicoli RM. 1951. Phlébotomes de la Corse cristalline. Etude biométrique et critique du *Phlebotomus perniciosus legeri* (J. Mansion, 1913) (Nematocera, Psychodidae). Bulletin de la Société de Pathologie Exotique, 44, 495-509.
109. Nicoli RM. 1952. Phlébotomes de la Corse : biologie du *Phlebotomus legeri* (Mansion, 1913). Comptes Rendus des Séances de la Société de Biologie et de ses Filiales, 146, 578-580.
110. Nicoli RM. 1963. Extension en France du *Larroussius ariasi* (A. Tonnoir, 1921) [Dipt. Nematocera]. Bulletin de la Société Entomologique de France, 68, 233-238.
111. Nicoli RM, Nicoli J. 1960. L'apparition des Phlébotomes adultes en Corse au cours de l'été (*Larroussius perniciosus* legeri) (J. Mansión, 1913) (Dipt. Nemat. Psychodidae). Données climatiques. Bulletin de la Société Entomologique de France, 61, 22-26.
112. Nitzulescu V, Dollfus R-P. 1934. Présence à Richelieu (Indre-et-Loire) de *Phlebotomus perniciosus* Newstead. Annales de Parasitologie Humaine et Comparée, 12(1), 67-69.
113. Parrot L. 1922. Présence de *Phlebotomus perniciosus* Newstead dans la région parisienne. Bulletin de la Société de Pathologie Exotique, 15, 694.
114. Parrot L. 1933. Notes sur les phlébotomes. V. Présence de *Phlebotomus ariasi* Tonnoir dans le sud-ouest de la France. Archives de l'Institut Pasteur d'Algérie, 11(4), 599-602.
115. Parrot L. 1936. Notes sur les phlébotomes. XXI. Sur la valve copulatrice de *Phlebotomus perniciosus* Newstead. Archives de l'Institut Pasteur d'Algérie, 14(2), 144-148.
116. Parrot L. 1936. Notes sur les phlébotomes. XXII. Présence de *Phlebotomus perniciosus* dans le département de l'Indre. Archives de l'Institut Pasteur d'Algérie, 14, 427.
117. Parrot L. 1943. Notes sur les phlébotomes, XL. Sur *Phlebotomus* (*Prophlebotomus*) *minutus* Rondani et sa variété *parroti* Adler et Theodor. Archives de l'Institut Pasteur d'Algérie, 21, 38-50.
118. Parrot L, Durand-Delacre R. 1952. Notes sur les phlébotomes. LXVI. Sur le mâle de *Phlebotomus mascittii* et sur sa présence en France. Archives de l'Institut Pasteur d'Algérie, 30(4), 393-397.
119. Perrotey S, Mahamdallie SS, Pesson B, Richardson KJ, Gállego Culleré M, Ready PD. 2005. Postglacial dispersal of *Phlebotomus perniciosus* into France. Parasite, 12(4), 283-291.
120. Pesson B, Leger N, Madulo-Leblond G, Petavy AF, Cambon M. 1985. La leishmaniose en Auvergne. Médecine et Maladies Infectieuses, 3, 107-109.
121. Pesson B, Wallon M, Floer MT, Kristensen AR. 1991. Étude isoenzymatique de populations méditerranéennes de phlébotomes du sous-genre *Larroussius*. Parassitologia, 33, 471-476.

122. Peyrefitte CN, Grandadam M, Bessaud M, Andry PE, Fouque F, Caro V, Diancourt L, Schuffenecker I, Pages F, Tolou H, Zeller H, Depaquit J. 2013. Diversity of *Phlebotomus perniciosus* in Provence, southeastern France: Detection of two putative new phlebovirus sequences. Vector-Borne and Zoonotic Diseases, 13(9), 630-636.
123. Poinsot S. 1972. Structure des populations de phlébotomes (Diptera -Psychodidae). Thesis. Université des sciences et techniques du Languedoc: Montpellier (France).
124. Pringault E. 1920. Phlébotomes de la région marseillaise. Bulletin de la Société de Pathologie Exotique, 13, 809-810.
125. Prudhomme J, Rahola N, Toty C, Cassan C, Roiz D, Vergnes B, Thierry M, Rioux J-A, Alten B, Sereno D, Banuls A-L. 2015. Ecology and spatiotemporal dynamics of sandflies in the Mediterranean Languedoc region (Roquedur area, Gard, France). Parasites and Vectors, 8(1), 642-655.
126. Prudhomme J, Toty C, Erisoz Kasap O, Rahola N, Vergnes B, Maia C, Campino L, Antoniou M, Jimenez M, Molina R, Cannet A, Alten B, Sereno D, Banuls A-L. 2015. New microsatellite markers for multi-scale genetic studies on *Phlebotomus ariasi* Tonnoir, vector of *Leishmania infantum* in the Mediterranean area. Acta Tropica, 142, 79-85.
127. Rageau J. 1948. Observations sur les phlébotomes de la région de Poitiers. Bulletin de la Société de Pathologie Exotique, 41, 217-222.
128. Ranque J, Quilici M, Dunan S. 1975. Les leishmanioses du Sud-Est de la France. Ecologie - Epidémiologie - Prophylaxie. Acta Tropica Separatum, 32(4), 371-380.
129. Ranque J, Quilici M, Dunan S. 1977. Les leishmanioses de la région provençale. Considérations épidémiologiques et écologiques, in: Ecologie des leishmanioses. Colloques lnternationaux du CNRS. CNRS: Paris (France). p. 285-293.
130. Raynal J. 1954. Les Phlébotomes de France et leur distribution régionale. Annales de Parasitologie Humaine et Comparée, 29(3), 297-323.
131. Raynal J, Le Gac P. 1932. Sur la présence de *Phlebotomus papatasi* Scopoli 1786 à Marseille. Annales de Parasitologie Humaine et Comparée, 10(6), 497-503.
132. Raynal J, Le Gac P. 1932. Sur un exemplaire de *Phlebotomus larroussei* Langeron et Nitzulescu 1931, capturé à la rose (banlieue de Marseille). Annales de Parasitologie Humaine et Comparée, 10(6), 504-508.
133. Raynal J, Le Gac P. 1933. Étude sur plusieurs lots de Phlébotomes capturés dans différents quartiers de Marseille. Annales de Parasitologie Humaine et Comparée, 11(4), 249-267.
134. Raynal J, Le Gac P. 1933. Note sur un Phlébotome du groupe *minutus* capturé à Sainte-Maxime (Var). Bulletin de la Société de Pathologie Exotique, XXVI, 455-458.
135. Raynal J, Le Gac P. 1933. Sur trois phlébotomes femelles capturés dans les Pyrénées à Capvern et pouvant être rapportés à *Phlebotomus ariasi* femelle. Bulletin de la Société de Pathologie Exotique, 26, 652-660.
136. Raynal J, Le Gac P. 1934. Présence de *Phlebotomus larroussei* à Capvern (Hautes-Pyrénées). Annales de Parasitologie Humaine et Comparée, 12(5), 343-344.
137. Raynal J, Le Gac P. 1934. Sur la présence de *Phlebotomus ariasi* mâle et femelle à Capvern. Bulletin de la Société de Pathologie Exotique, 27, 467-473.
138. Ready PD, Croset H. 1980. Diapause and laboratory breeding of *Phlebotomus perniciosus* Newstead and *Phlebotomus ariasi* Tonnoir (Diptera: Psychodidae) from southern France. Bulletin of Entomological Research, 70(3), 511-523.
139. Ready PD, Ready PA. 1981. Prevalence of *Phlebotomus* spp. in southern France: sampling bias due to different man-biting habits and autogeny. Annals of Tropical Medicine & Parasitology, 75(4), 475-476.
140. Ready PD, Smith DF, Killick-Kendrick R. 1988. DNA hybridizations on squash-blotted sandflies to identify both *Phlebotomus papatasi* and infecting *Leishmania major*. Medical and Veterinary Entomology, 2, 109-116.
141. Rioux J-A, Abonnenc E, Baudouy JP. 1965. Un cas de gynandromorphisme chez *Phlebotomus ariasi* Tonnoir [Dipt. Psychodidae]. Annales de la Société Entomologique de France, 3, 615-617.
142. Rioux J-A, Carron S, Dereure J, Perieres J, Zeraia L, Franquet E, Babinot M, Gallego M, Prudhomme J. 2013. Ecology of leishmaniasis in the South of France. 22. Reliability and representativeness of 12 *Phlebotomus ariasi*, *P. perniciosus* and *Sergentomyia minuta* (Diptera: Psychodidae) sampling stations in Vallespir (eastern French Pyrenees region). Parasite, 20, 34-44.
143. Rioux J-A, Croset H, Aboukler JP, Papierok B. 1972. Écologie des leishmanioses dans le sud de la France. 4. Infestation d'une population naturelle de *Phlebotomus ariasi* Tonnoir, 1921. Annales de Parasitologie Humaine et Comparée, 47(2), 325-330.
144. Rioux J-A, Croset H, Houin R, Papierok B, Tour S. 1971. Observations sur les hauteurs de vol de *Phlebotomus ariasi* Tonnoir, 1921. Annales de Parasitologie Humaine et Comparée, 46(3), 277-283.
145. Rioux J-A, Croset H, Maistre M. 1974. Un cas de gynandromorphisme chez *Sergentomya minuta* (Rondani, 1843) (Diptera-Psychodidae). Annales de Parasitologie Humaine et Comparée, 49(3), 371-372.
146. Rioux J-A, Golvan Y-J, Houin R, Deltour F. 1962. Nouvelles stations françaises de *Phlebotomus ariasi* Tonnoir 1921, *P. perniciosus* Rondani 1843, *P. mascitti* Grassi 1908, et *P. minutus* Rondani 1843. Annales de Parasitologie Humaine et Comparée, 37(4), 630-632.
147. Rioux J-A, Golvan Y, Croset H, Tour S, Houin R, Abonnenc E, Petitdidier M, Vollhardt Y, Dedet JP, Albaret JL, Lanotte G, Quilici M, Martini-Dumas A, Maistre M, Brès A, Roviralta T, Vila F. 1969. Épidémiologie des leishmanioses dans le Sud de la France. Vol. 37. Paris (France): Monographie de l'Institut National de la Santé et de la Recherche Médicale. 228 pp.
148. Rioux J-A, Golvan YJ, Maistre O. 1961. Présence de *Phlebotomus* (*Larroussius*) *ariasi* Tonnoir 1921 dans les départements de l'Aveyron, des Bouches-du-Rhône, du Gard, de l'Hérault, des Pyrénées-Orientales et du Vaucluse. Annales de Parasitologie Humaine et Comparée, 36, 706-707.
149. Rioux J-A, Houin R, Baudouy J, Croset H, Tour S. 1970. Présence en Corse de *Phlebotomus* (*Paraphlebotomus*) *sergenti* Parrot, 1917. Annales de Parasitologie Humaine et Comparée, 45(3), 343-356.

150. Rioux J-A, Houin R, Leger N, Croset H, Deniau M, Poinsot S. 1971. Nouvelles stations Corses de *Phlebotomus sergenti* Parrot, 1917. Annales de Parasitologie Humaine et Comparée, 46(3), 329-336.
151. Rioux J-A, Jarry D, Lanotte G, Maazoun R, Killick-Kendrick R. 1984. Écologie des leishmanioses dans le sud de la France. 18. Identification enzymatique de *Leishmania infantum* Nicolle, 1908, isolé de *Phlebotomus ariasi* Tonnoir, 1921 spontanément infesté en Cévennes. Annales de Parasitologie Humaine et Comparée, 59(4), 331-333.
152. Rioux J-A, Jarry D, Maazoun R, Wallbanks K. 1982. Confirmation de l'existence en France continentale de *Phlebotomus sergenti* Parrot, 1917. Annales de Parasitologie Humaine et Comparée, 57(6), 647-648.
153. Rioux J-A, Killick-Kendrick R, Leaney A, Turner D, Bailly M, Young C. 1979. Écologie des leishmanioses dans le Sud de la France. 12. Dispersion horizontale de *Phlebotomus ariasi* Tonnoir, 1921. Expériences préliminaires. Annales de Parasitologie Humaine et Comparée, 54(6), 673-682.
154. Rioux J-A, Killick-Kendrick R, Perieres J, Turner D, Lanotte G. 1980. Écologie des leishmanioses dans le sud de la France. 13. Les sites de "flanc de coteau", biotopes de transmission privilégiés de la leishmaniose viscérale en Cévennes. Annales de Parasitologie Humaine et Comparée, 55(4), 445-453.
155. Rioux J-A, Lanotte G, Croset H, Dedet J-P. 1972. Écologie des leishmanioses dans le sud de la France. 5. Pouvoir infestant comparé des diverses formes de Leishmaniose canine vis-à-vis de *Phlebotomus ariasi* Tonnoir, 1921. Annales de Parasitologie Humaine et Comparée, 47(3), 413-419.
156. Rioux J-A, Lanotte G, Perieres J, Croset H. 1973. Écologie des leishmanioses dans le sud de la France. 6. Première mention de l'infestation spontanée de *Phlebotomus ariasi* Tonnoir, 1921. Annales de Parasitologie Humaine et Comparée, 48(4), 519-522.
157. Rioux J-A, Lanotte G, Pratlong F, Dereure J, Jarry D, Moreno G, Killick-Kendrick R, Perieres J, Guilard E, Belmonte A, Portus M. 1985. La leishmaniose cutanée autochtone dans le Sud-Est de la France. Résultats d'une enquête éco-épidémiologique dans les Pyrénées-Orientales. Médecine et Maladies Infectieuses, 11, 650-656.
158. Ristorcelli A. 1936. Sur la présence à Toulouse de *Phlebotomus perniciosus*. Archives de l'Institut Pasteur d'Algérie, 14, 426.
159. Ristorcelli A. 1939. Présence à Melun (Seine-et-Marne) de *Phlebotomus perniciosus*. Annales de Parasitologie Humaine et Comparée, 17(4), 374.
160. Roman E, Coudert J. 1943. Les Phlébotomes à Lyon et dans la banlieue lyonnaise. Journal de Médecine de Lyon, 185-186.
161. Rossi P. 1926. Contribution à l'étude du phlébotome en Aunis. Bulletin de la Société de Pathologie Exotique, 19(8), 705-709.
162. Rossi P. 1935. Sur la présence de *Phlebotomus perniciosus* à Mâcon. Bulletin de la Société de Pathologie Exotique, 28, 282-284.
163. Schaffner F. 2023. Occurrence of *Phlebotomus mascittii* (Diptera: Psychodidae: Phlebotominae) in the Upper Rhine Valley of Alsace, France. Annales de la Société Entomologique de France, 59(4), 278-284.
164. Séguy E. 1924. Diptera: Recueil d'études biologiques et systématiques sur les diptères du globe. Vol. 1. Paris (France): Paul Lechevalier. 135 pp.
165. Senevet G. 1935. *Phlebotomus perniciosus* en France. Bulletin de la Société de Pathologie Exotique, 28, 581.
166. Sicart M. 1954. Récoltes de Phlébotomes en France. Bulletin de la Société d'Histoire Naturelle de Toulouse, LXXXIX, 235.
167. Sicart M, Escande F, Ruffié J. 1958. Les Psychodidés du sous-bassin aquitain. Bulletin de la Société d'Histoire Naturelle de Toulouse, XCIII, 70-72.
168. Toumanoff C, Chassignet R. 1954. Contribution à l'étude des phlébotomes en Corse. Bulletin de l'Institut National d'Hygiène, 9(3), 664-687.
169. Vermeil C. 1949. Localité nouvelle pour *Phlebotomus perniciosus*. Annales de Parasitologie, 24(3-4), 396.
170. Villeneuve J. 1909. À propos du *Phlebotomus papatasi* Scop. [Dipt.]. Bulletin de la Société Entomologique de France, 14(11), 195-196.
171. Ward RD, Pasteur N, Rioux J-A. 1981. Electrophoretic studies on genetic polymorphism and differentiation of phlebotomine sandflies (Diptera: Psychodidae) from France and Tunisia. Annals of Tropical Medicine & Parasitology, 75(2), 235-245.
172. Wilkes TJ, Rioux J-A. 1980. The application of Polodova's technique for the age determination of *Phlebotomus (Larroussius) ariasi*. Transactions of the Royal Society of Tropical Medicine and Hygiene, 74, 119.
173. Young CJ, Turner DP, Killick-Kendrick R, Rioux J-A, Leaney AJ. 1980. Fructose in wild-caught *Phlebotomus ariasi* and the possible relevance of sugars taken by sandflies to the transmission of leishmaniasis. Transactions of the Royal Society of Tropical Medicine and Hygiene, 74(3), 363-366.
